# *Ang1* and *Ang4* differentially affect colitis and carcinogenesis in an AOM-DSS mouse model

**DOI:** 10.1371/journal.pone.0281529

**Published:** 2023-03-07

**Authors:** Alexander Hu, Cullen Roberts, Andrei Moscalu, Mark Redston, James Yoo

**Affiliations:** 1 Department of Surgery, Harvard Medical School, Brigham and Women’s Hospital, Boston, Massachusetts, United States of America; 2 Department of Pathology, Harvard Medical School, Brigham and Women’s Hospital, Boston, Massachusetts, United States of America; University of Lille, FRANCE

## Abstract

**Introduction:**

Angiogenin-1 (*Ang1)* and angiogenin-4 (*Ang4)* are 14-kDa ribonucleases with potent angiogenic and antimicrobial properties. The role of *Ang1* and *Ang4* in chronic colitis and colitis-associated cancer has not been previously studied.

**Methods:**

Wild-type (WT) and angiogenin-1 knock-out (*Ang1-*KO) C57BL/6 mice were given azoxymethane, a colon carcinogen, 2 days in advance of three cycles of 3.5% dextran sodium sulfate (DSS). Disease activity index (DAI) was recorded, a colonoscopy was performed after each DSS treatment, and mice were euthanized (colitis, recovery, cancer) with tissue evaluated by histopathology. *Ang1*, *Ang4*, TNF-α, *Il-1F062*, *IL-6*, *IL-10*, *IL-23*, *IL-33* mRNA levels were analyzed by RT-PCR.

**Results:**

*Ang1-*KO mice exhibited more severe colitis compared to WT mice during both the acute (P<0.05) and recovery (P<0.05) phases of each DSS cycle. Consistent with these results, colonic TNF-α, IL1-β, IL-6, IL-10, and IL-33 mRNA levels were significantly upregulated in *Ang1-*KO mice (P<0.05). While *Ang4* increased to similar levels in both WT and *Ang1*-KO mice during colitis and recovery phases, WT mice were distinguished by a significant upregulation of *Ang1*. Interestingly, despite the reduced colitis, WT mice developed significantly more tumors compared to *Ang1-*KO mice (P<0.05). 134 tumors formed in WT mice (4.6 tumors/mouse) while only 46 tumors formed (1.5 tumors/mice) in *Ang1-*KO mice, which were also characterized by a 34-fold decrease in *Ang4* compared to WT mice and the complete absence of *Ang1*.

**Conclusions:**

In a mouse model of colitis-associated cancer, *Ang1-*KO mice develop more severe colitis, but fewer tumors compared to WT mice. *Ang1* levels correlate with the severity of colitis and the development of colitis-associated cancer, while *Ang4* was upregulated during both colitis and cancer. *Ang1* and *Ang4* play important regulatory roles in the response to chronic colitis and the development of colitis-associated cancer and may serve as novel therapeutic targets.

## Introduction

One of the most serious consequences of chronic inflammatory bowel disease (IBD) is the development of colorectal cancer, which can occur in up to 18% of patients [1]. Colitis-associated cancer is characterized by rapid progression and high mortality compared to sporadic forms [2–4]. Surveillance programs designed to detect precursor dysplasia may overtreat low-risk patients but may miss interval cancers in high-risk patients, highlighting the need to develop new prevention and treatment strategies. While the association between chronic inflammation and cancer is well established, the precise mechanism(s) leading to intestinal neoplasia remains unclear.

In this context, we have been interested in the role of angiogenin, an endogenous antimicrobial peptide [5–7] that is highly expressed in the GI tract and known to be induced by microbial colonization [8–10]. Angiogenin has garnered increasing attention for its role in IBD. Human patients with active IBD have been noted to have elevated serum levels of angiogenin [11, 12], and we and others have found that angiogenin expression is not only altered in the setting of colitis [6–8, 13], but is a key modulator of the physiologic responses of the GI microenvironment to inflammation [6, 14]. Bai et al found that colonic expression of angiogenin is decreased in human patients with active ulcerative colitis and Crohn’s disease, and that angiogenin is protective against acute DSS colitis in mice [6]. Specifically, exposure of angiogenin-knockout (*Ang1*-KO) mice to 2.5% DSS over 7 days led to greater mucosal permeability and weight loss, a higher disease activity index, and shorter colon length compared to wild type (WT) mice, suggesting that angiogenin plays an important role in the response to acute colitis.

Work by Sun et al. confirmed that both mouse and human angiogenin demonstrate antimicrobial activity, and that *Ang1* deficiency in mice leads to significant changes in the microbiome that regulates the response to acute colitis [7]. *Ang1* deficiency resulted in a gut dysbiosis, notable for up-regulation of colitogenic α-Proteobacteria and down-regulation of protective Lachnospiraceae, which correlated with the degree of colitis following exposure to 2.5% DSS. Strikingly, and consistent with data from Bai et al. [6], they found that administration of exogenous *Ang1* both restores the microbiome and mitigates DSS-induced colitis, demonstrating that angiogenin has influential antimicrobial actions that can be utilized therapeutically.

In summary, an accumulating body of evidence suggests that angiogenin has important effects on the gut microbiome and is dynamically regulated in the setting of acute colitis. However, the impact of angiogenin on chronic colitis and the development of colitis-associated cancer has not been previously studied. Also, prior studies did not account for angiogenin-4 (*Ang4*), another angiogenin sub-type that is highly expressed in the murine GI tract. The goal of our present study was to explore the role of both angiogenin-1 and angiogenin-4 in the setting of chronic colitis and colitis-associated cancer in a murine model. Here, we report for the first time that angiogenin-1 and angiogenin-4 are differentially regulated in this setting and correlate with dramatic differences in the degree of colitis and the development of colitis-associated cancer.

## Materials and methods

The study was approved by the Brigham and Women’s Hospital IACUC (#2019N000208).

### Mice

Angiogenin-1 knockout (*Ang1*-KO) mice were generously provided by Guo-fu Hu from Tufts Medical Center, Boston MA. Male and female eight- to ten-week-old *Ang1-*KOmice and their wild-type (WT) control were housed in a climate-controlled environment with a 12-h light/dark cycle, at 68–75⁰F and 35–65% humidity, while reared on a regular chow diet with ad libitum access to water unless noted otherwise. The WT group consisted of 15 male mice and 16 female mice. The *Ang1-*KO group consisted of 15 male mice and 16 female mice. Mice were sacrificed by isoflurane anesthetic overdose followed by cervical dislocation. Mice were closely monitored for signs of distress, were provided supportive care (i.e. food and water, diet gel, hydrogel and/or subcutaneous fluids) as needed during the critical colitis phase, and were euthanized if they showed signs of an adverse reaction suggesting pain or discomfort (lethargy, weight loss of greater than 15%, persistent diarrhea or bloody stool for more than 3 days, moderate or severe rectal prolapse, labored breathing, peritonitis or bowel perforation). All animal studies were performed in compliance with the guidelines set by the protocol approved by the Institutional Animal Care and Use Committee.

### AOM-DSS

Azoxymethane (AOM, 10mg/kg, Sigma-Aldrich- A5486-25MG) in saline was injected intraperitoneally (IP) 2 days in advance of three cycles of 3.5% dextran sodium sulfate (DSS, MP Biomedicals- 160110, molecular weight: 36,000–50,000). Each cycle consisted of 5 days of 3.5% DSS in the drinking water, followed by 16 days of regular water. Mice were euthanized at various time points (“baseline”; at the end of each DSS treatment, designated as “colitis”; one week after each DSS cycle, designated as “recovery”, with the end of the 3^rd^ cycle being designated as “cancer”). This AOM-DSS colitis model leads to predictable and reproducible colitis and cancer [15]. Disease activity index (DAI) was recorded, a colonoscopy was performed 1 week after each DSS treatment, and mice were euthanized at various time points (colitis, recovery, cancer) with colon tissue either stored in liquid nitrogen or placed in formalin. Disease activity index was calculated based on weight loss (1 = 0–10%, 2 = 10–15%, 3 = 15–20%, 4 = >20%), stool consistency (1 = solid, 2 = loose, 3 = wet, 4 = diarrhea), and hematochezia (1 = no blood, 2 = slight blood, 3 = blood, 4 = significant hematochezia).

### Mouse colonoscopy

Anesthesia was induced by using a mix of 95% O2 and isoflurane 3%, followed by maintenance using a mix of 95%O2 and 1.5–3% isoflurane, all at a rate of 1000ml/min and colonoscopies were performed with a 1.9mm Karl Storz Coloview mini-endoscopic system. The colonoscope was passed into the distal colon under direct endoscopic visualization. Colonoscopies were performed on days 14, 35, and 56 with video documentation.

### Histopathological analysis

Mouse colons were Swiss-rolled and preserved in 10% formalin. Slides were prepared by the Rodent Histopathology [Dana Farber/Harvard Cancer Center] core at Harvard Medical Center using hematoxylin and eosin (H&E) staining. Histologic analysis was performed by a blinded, board-certified GI pathologist for tumor number and colitis severity. Colitis scores were based on the following factors: ‘inflammatory infiltrates’, ‘goblet cell loss’, ‘hyperplasia’, ‘crypt density’, ‘muscle thickness’, ‘submucosal infiltration’, ‘ulcerations’ and ‘crypt abscesses’ (with grades defined as the following: grade 0 = normal; grade 1 = mucosal changes with minimal ulceration; grade 2 = significant reactivity and inflammation without ulceration; grade 3 and 4 = ulceration) as described in Koelink et al [16].

### qRT-PCR

Relative mRNA expression levels (*Ang1*, *Ang4*, TNF-α, IL1-β, IL-6, IL-10, and IL-33*)* were measured using quantitative real-time PCR. Total RNA from colon tissue was extracted using a Qiagen RNeasy mini Kit (Qiagen- 74104). GAPDH was the reference gene used. Following DNase treatment (Qiagen- 79254), cDNA was synthesized using a High-Capacity cDNA Reverse Transcription Kit (Thermofisher-4368814). Transcript levels were quantified using a SYBR Green reporter (Applied Biosystems- 4309155) in an Applied Biosystems 7300 real time PCR system (Applied Biosystems). The primer sequences are: *Ang1* Fwd: CAT CCC AAC AGG AAG GAA GGA; *Ang1* Rev: ACC TGG AGT CAT CCT GAG CC. *Ang4* Fwd: GGC ACC AAG AAA AAC ATC AGG GC; *Ang4* Rev: GTG CGT ACA AGT GGT GAT CTG G.

### 16S rRNA sequencing

Stool samples from WT and *Ang1*-KO mice were collected and sent to the Massachusetts Host-Microbiome Center. DNA was extracted, amplified, and fecal microbiota DNA was extracted using QIAamp Fast DNA Stool Mini Kit (Qiagen, Germany). Variable region 4 (v4) of the 16S rRNA gene was amplified on the Illumina MiSeq platform. All microbiome analyses were done by an independent third party, Harvard Chan Microbiome Analysis (HCMA) Core (https://hcmph.sph.harvard.edu/hcmac/).

### Statistical analysis and power calculation

Disease activity index belongs to the type of “Quantitative, Continuous Non-normal” data, and were analyzed by “Mann-Whitney U/Wilcoxon Rank Sum Test” for two groups, and “Kruskal-Wallis Test” for experiments involving more than two groups. Tumor number and sizes are “Continuous Normal Quantitative” data and were analyzed by t-test (between two groups) or ANOVA (more than two groups). IHC score, lesion severity, and tumor grade are “Categorical, Ordinal” data and were analyzed by “Fisher’s exact test for small samples (Z test)” when two groups are compared, or by “Chi square test” when more than two groups are compared. Quantitative data was presented as means ± standard deviation (SD). A *P* value of <0.05 is considered to be significant. Power calculation indicates that ten mice per group are needed to detect a 2 SD difference using a two-tailed 5% significance level at 80% power.

## Results

### *Ang1*-deficient mice develop more severe DSS-induced colitis

**Fig 1:**
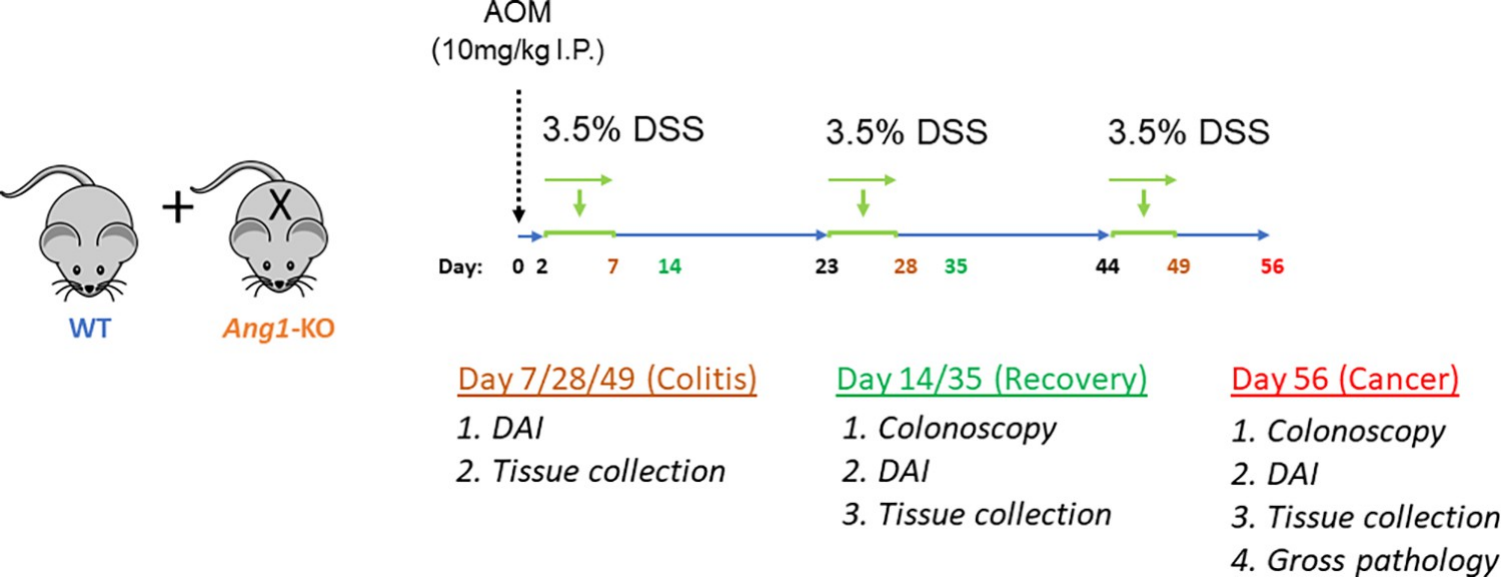
Experimental protocol. WT and *Ang1*-KO mice were given azoxymethane (AOM) followed by 3 cycles of 3.5% DSS. Disease activity index (DAI) was recorded, a colonoscopy was performed, and tissue was collected at the times indicated.

**Fig 2:**
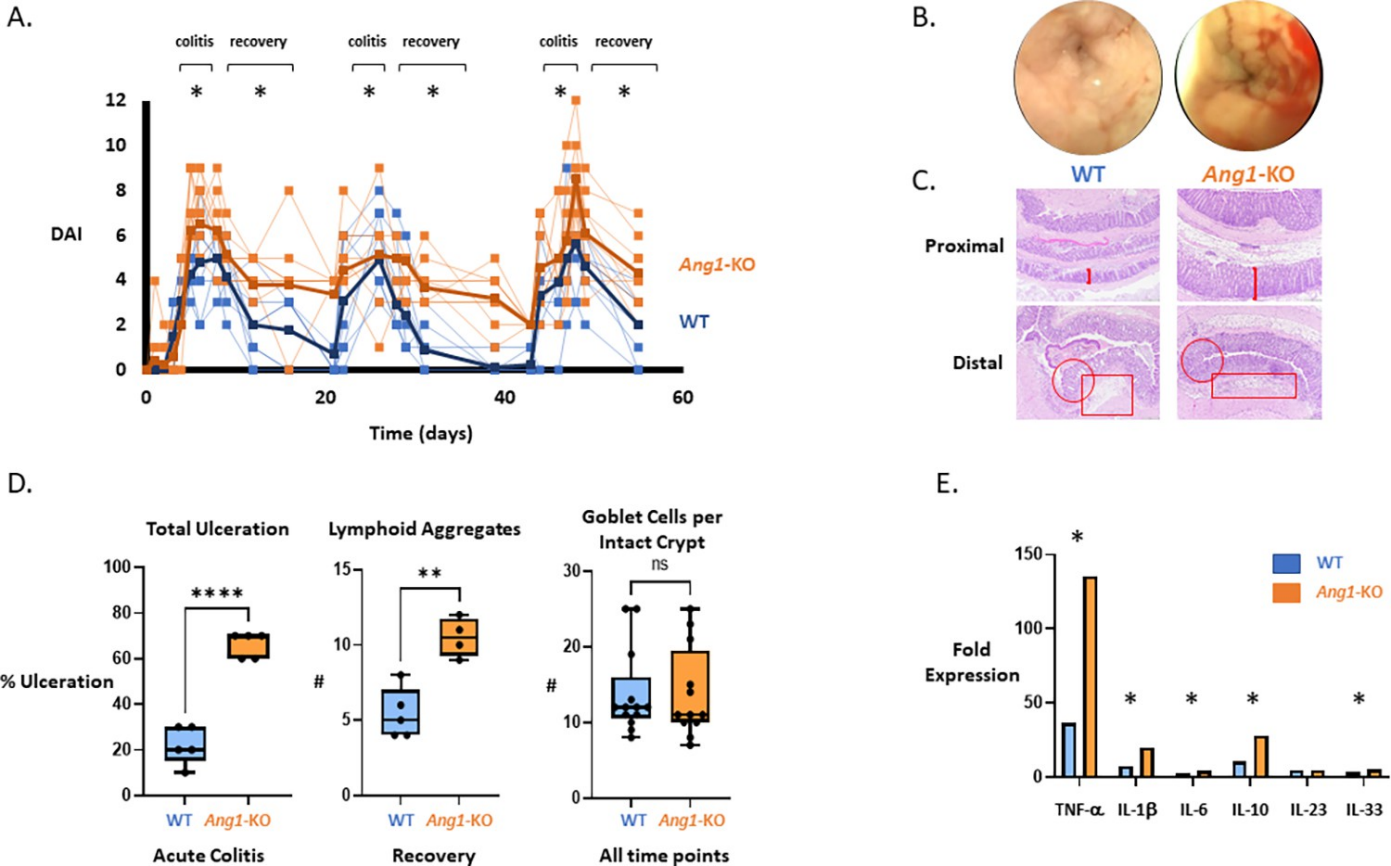
*Ang1*-KO mice develop more severe colitis compared to WT mice. **A**.DAI was recorded after each DSS cycle. * denotes statistical significance (P<0.05). **B**. Representative images of the endoscopic view one week after exposure to DSS. **C**. Histologic analysis of colon tissue after DSS. In the proximal colon, the mucosa (arrows) is thicker in *Ang1*-KO mice, with longer crypts in a more regenerative, inflamed, and reactive state. In the distal colon, *Ang1*-KO mice are notable for an absence of crypts (circles) and greater ulceration, with increased immune cells in the lamina propria compared to WT mice (rectangles). **D**. Histologic analysis demonstrated a significant increase in total ulceration and lymphoid aggregates in *Ang1*-KO mice, with no difference in goblet cells per intact crypt compared to WT mice. **** denotes P<0.0001, ** denotes P<0.005. **E**. During acute colitis, mouse colon tissue from *Ang1*-KO mice had significantly higher mRNA expression of TNF-α, IL-1β, IL-6, IL-10, and IL-33. * denotes statistical significance (P<0.05).

Wild-type (WT; n = 29) and angiogenin-1 knock-out (*Ang1-*KO; n = 31) C57BL/6 mice, a whole-body homozygous knockout strain, were given azoxymethane (AOM, 10mg/kg IP), a colon carcinogen, two days in advance of three cycles of 3.5% dextran sodium sulfate (DSS) (**Fig 1**). During the acute phase of the first DSS cycle, *Ang1-*KO mice exhibited more severe colitis with a significantly higher DAI compared to WT mice (**Fig 2A,** P<0.05), which correlated with the endoscopic findings and histologic analysis. While this result was consistent with prior studies, it was unknown whether this difference persisted with repeated exposure to DSS. We found that this difference was sustained during the acute (P<0.05) phase of the second and third DSS cycle as well. Most notably, we found that the difference in colitis severity was even more pronounced during the recovery phases of each cycle of DSS-induced colitis (**Fig 2A,** P<0.05), suggesting that angiogenin may be most relevant during the reparative processes that follow acute injury. We also found no difference in the degree of colitis, as measured by DAI, during both acute colitis and the recovery phase between male and female mice (data not shown). Mouse colonoscopies performed 1 week after each DSS cycle were subjectively notable for greater mucosal friability and gross blood in the *Ang1-*KO mice (**Fig 2B**). Given the relatively subjective nature of DAI and endoscopic appearance, the degree of colitis was confirmed by measuring inflammatory cytokine expression and by histologic examination. Histologically, *Ang1-*KO mice demonstrated more prominent and confluent ulcerations during both acute colitis and recovery (grade 2–4, P<0.05), more lymphoid aggregates (P<0.05), but no difference in the number of goblet cells per intact crypt (**Fig 2C and 2D**). Likewise, colonic TNF-α, IL1-β, IL-6, IL-10, and IL-33 mRNA levels, analyzed by RT-PCR, were significantly upregulated in *Ang1-*KO mice during recovery (**Fig 2E**).

### *Ang4* expression is altered in *Ang1*-deficient mice

**Fig 3:**
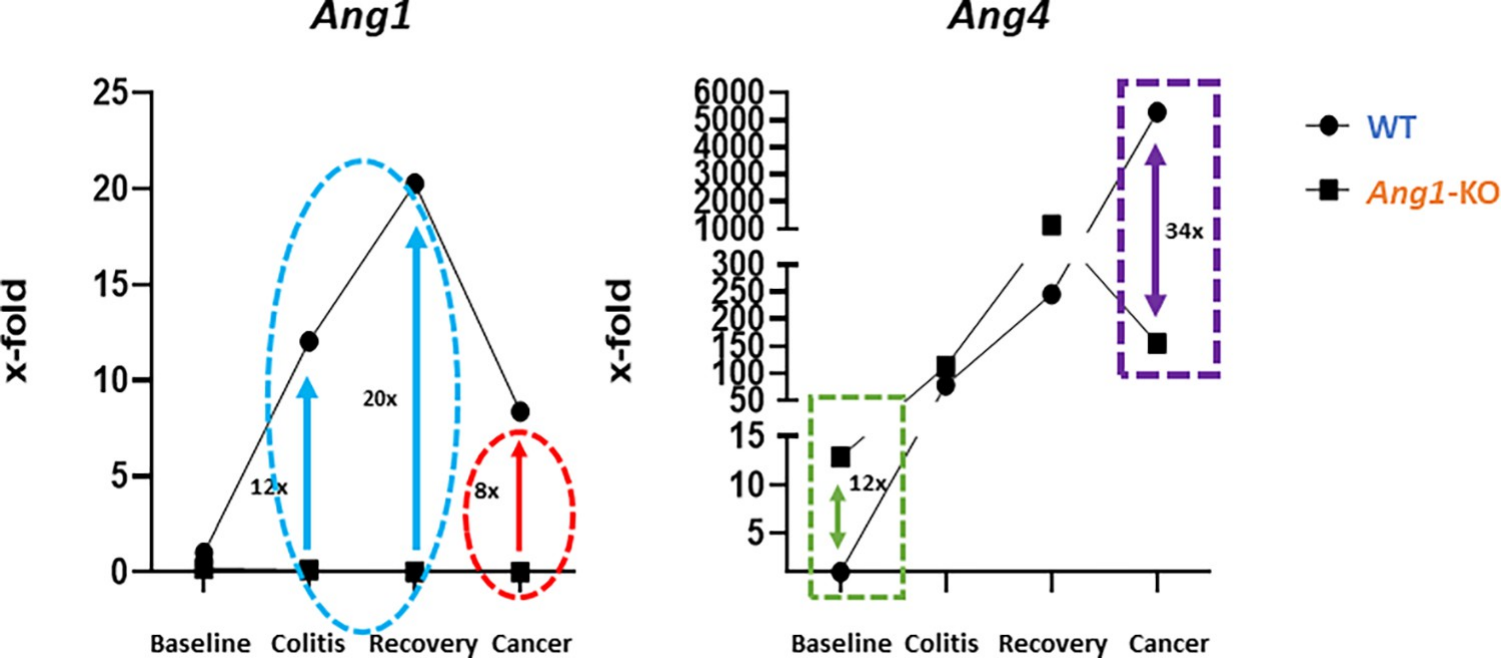
Differential *Ang1* and *Ang4* mRNA expression following AOM-DSS exposure. mRNA expression of *Ang1* and *Ang4* was analyzed by qRT-PCR from colon tissue at various time points. *Ang1*-KO mice demonstrated a 12x increase in *Ang4* expression (dashed green rectangle) compared to WT mice at baseline. *Ang1* mRNA expression was upregulated 12-fold during acute colitis and 20-fold during recovery compared to its baseline level (dashed blue circle). In the presence of cancer in WT mice, there was upregulation of both *Ang1* (8-fold compared to baseline, dashed red circle) and *Ang4* (34-fold compared to *Ang1*-KO mice, dashed purple rectangle). Circles represent fold change in WT mice compared to its own baseline. Rectangles represent fold change in *Ang4* comparing WT and *Ang1*-KO mice.

In WT mice, *Ang1* is highly expressed in the colon and liver [7], suggesting an important role in GI homeostasis. In mice, other angiogenin subtypes exist (*Ang2*, *Ang3*, *Ang4*, *Ang5*, *Ang6*) and a major limitation of prior studies has been a failure to account for these other subtypes. While the physiologic significance of *Ang2*, *Ang3*, *Ang5* and *Ang6* is not well understood, *Ang4* is the most well-studied, is also highly expressed in the colon [7], and has well-established and distinct antimicrobial properties [8, 9, 17–19]. We hypothesized that the effects that were attributed to *Ang1* in other studies may be partially attributable to *Ang4*. To evaluate the potential role of *Ang1* and *Ang4* on the response to colitis, mRNA expression was analyzed by qRT-PCR in WT and *Ang1-*KO mice at baseline, after exposure to DSS (colitis) and one week after exposure to DSS (recovery). As expected, *Ang1-*KO mice, which are phenotypically and histologically normal at baseline, do not express *Ang1* in any organ system (S1 Data). Interestingly, at baseline we found a 12-fold upregulation of *Ang4* expression in *Ang1-*KO mice compared to WT mice (**Fig 3,** dashed green rectangle). While other groups have reported that fecal [6, 7] angiogenin levels are reduced in the setting of murine colitis, angiogenin-4 levels within colon tissue have not been previously reported. We found that *Ang4* was upregulated in both WT and *Ang1*-KO mice during both the acute and recovery phases of colitis. However, we found that WT mice, which develop less severe colitis, were distinguished by a significant upregulation of *Ang1* within colon tissue during both acute colitis (12-fold) and recovery *(*20-fold) compared to its baseline level (**Fig 3**, dashed blue circle).

### *Ang1*-deficient mice develop fewer tumors following AOM-DSS

**Fig 4:**
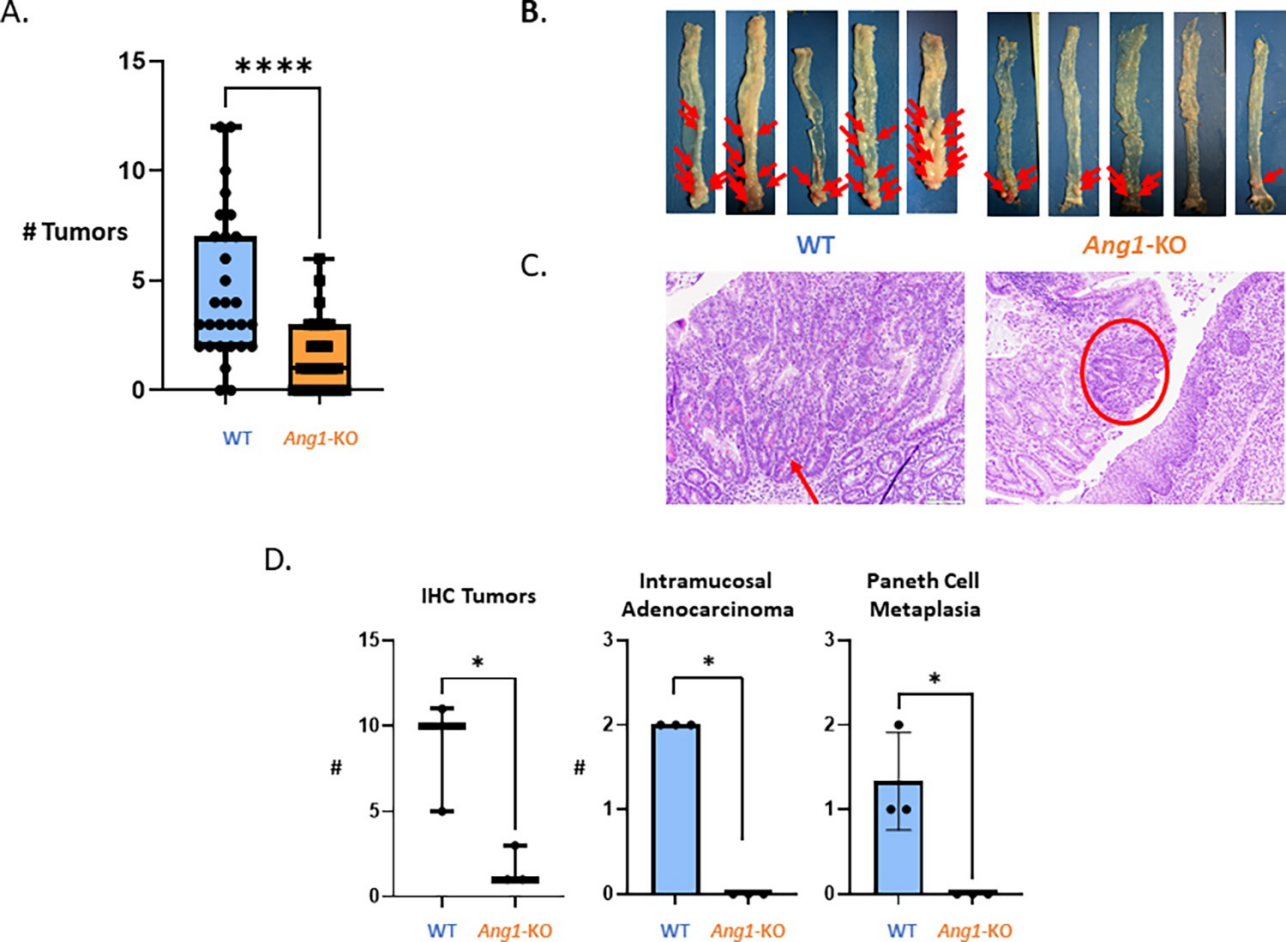
*Ang1*-deficient mice develop significantly fewer tumors compared to WT mice following AOM-DSS. **A**. Colon tissue was examined macroscopically, and the number of tumors was counted. **** denotes P<0.0001. **B**. Mouse colon tissue from representative experiments demonstrating the number and location of colon tumors (arrow heads). **C**. Representative immunohistochemical analysis of distal colon tissue from WT and *Ang1*-KO mice. WT mice demonstrated Paneth cell metaplasia (arrow) and intramucosal adenocarcinoma, while tumors in *Ang1*-KO were considerably smaller (circle). **D**. The number of tumors was evaluated by immunohistochemistry in Swiss-rolled mouse colon from WT (n = 3) and *Ang1*-KO (n = 3) mice following AOM-DSS. Tumors in WT mice were distinguished by the presence of intramucosal adenocarcinoma and Paneth cell metaplasia. * denotes statistical significance (P<0.05).

The most compelling and novel data we found is that despite the reduced colitis, WT mice developed significantly more tumors (P<0.05) compared to *Ang1-*KO mice at the end of the 3^rd^ DSS cycle (**Fig 4A**). On gross examination of mouse colon tissue, 134 tumors formed in WT mice (n = 29; 4.6 tumors/mouse) while only 46 tumors formed (n = 31; 1.5 tumors/mice) in *Ang1-*deficient mice (**Fig 4A and 4B**). We found no difference in tumor number comparing male and female mice (data not shown). This difference in tumor number was more pronounced when confirmed microscopically, by counting the number of histologically visible tumors present in Swiss-rolled colon (**Fig 4D**). In addition to having a higher tumor number, comparing distal tumors in WT and *Ang1*-KO mice, tumors in WT mice were larger, had a greater abundance of intramucosal adenocarcinoma compared to adenomas, and demonstrated Paneth cell metaplasia (**Fig 4C and 4D**). In the setting of cancer, WT mice were characterized by an 8-fold increase in *Ang1* compared to its baseline level and a 34-fold increase in *Ang4* (**Fig 3**, dashed purple rectangle) compared to *Ang1-*deficient mice.

### *Ang1*-deficient mice are associated with alterations in the gut microbiome

**Fig 5:**
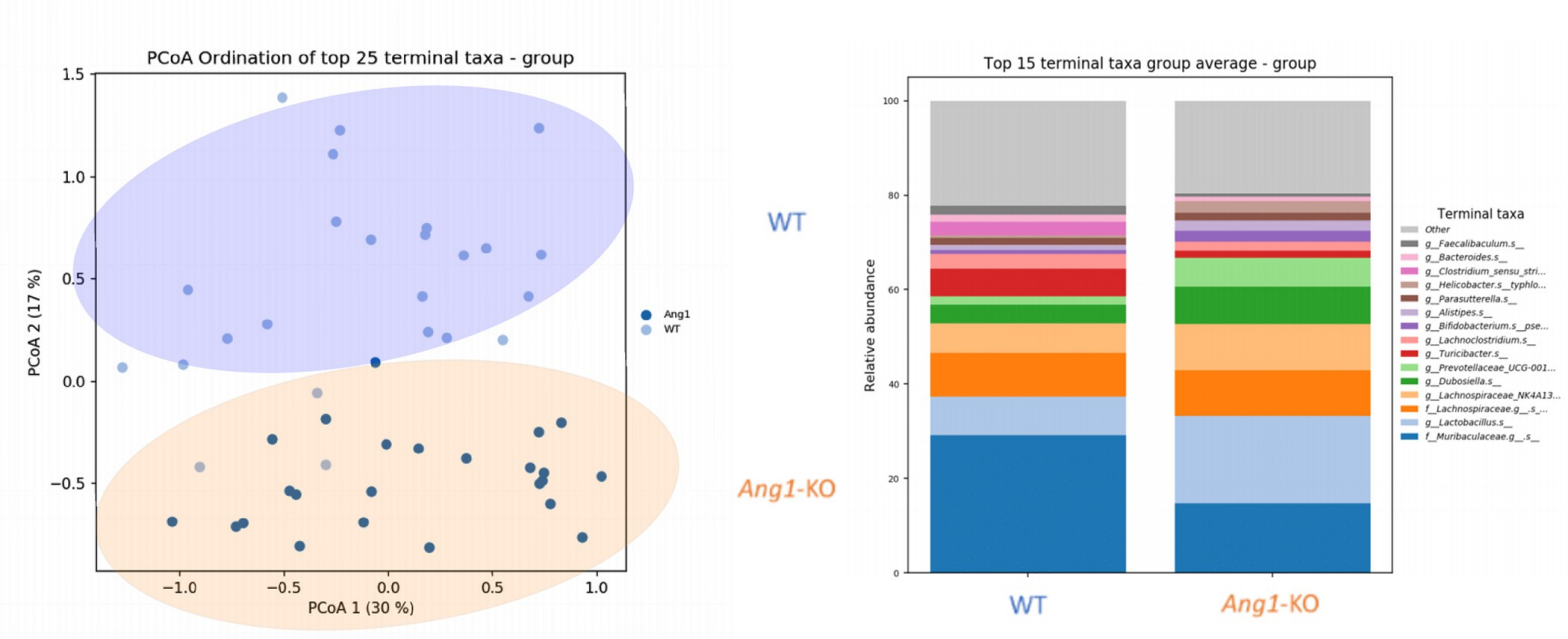
*Ang1*-deficient mice are associated with alterations in the gut microbiome. **A**. PCoA visualization of Bray-Curtis dissimilarities shows significant differences in the overall microbiome community between WT and *Ang1*-KO mice at baseline, confirmed by PERMANOVA (P < 0.001). **B**. Relative abundances of the top 15 terminal taxa. Multiple important features are seen to differ across phenotypes and are significant at FDR < 0.2 via MaAsLin testing framework.

To explore whether the effects of angiogenin-1 and angiogenin-4 on colitis and colitis-associated cancer are due to their anti-microbial properties, we began by performing 16S rRNA sequencing of stool comparing WT and *Ang1*-KO mice. At baseline, we showed that *Ang1*-KO mice do not express *Ang1* and have a 12-fold upregulation of *Ang4* compared to WT mice. In this setting, we identified dramatic community level differences between WT and *Ang1*-KO mice comparing the top 15 terminal taxa (**Fig 5**). Notably we identified a significant downregulation of Lachnospiraceae in *Ang1*-KO mice (data not shown), a finding that was consistent with prior studies [7]. Also notable is that despite repeated exposure to DSS, the distinct differences in community composition were sustained during recovery from colitis and in the presence of cancer (data not shown).

## Discussion

Utilizing the AOM-DSS mouse model of colitis-associated cancer, the present study suggests that angiogenin-1 and angiogenin-4affect the response to both acute and chronic colitis as well as the development of colitis-associated cancer. Specifically, we found that *Ang1-*KO mice, a whole-body homozygous knockout strain, develop more severe colitis but interestingly, significantly fewer tumors compared to wild-type (WT) mice. This discordance between the severity of colitis and the development of cancer was surprising and suggests that angiogenin-specific alterations in the colonic microenvironment play an important mechanistic role.

Prior studies have examined phenotypic differences following exposure to DSS in *Ang1*-KO mice [6, 7] but did not evaluate the expression of other angiogenin subtypes, along with their potential contributions to the altered phenotype. Within this context, we identified significant alterations in the expression of *Ang4*, another angiogenin subtype that is highly expressed in the mouse colon [7] and has well-established and distinct antimicrobial effects [8, 9, 17, 19]. Upon deletion of *Ang1*, *Ang4* is upregulated at baseline, suggesting an important role of *Ang4* in the absence of *Ang1*. While *Ang4* expression increased during colitis in both WT and *Ang1*-KO mice, there was a dramatic upregulation of *Ang4* in WT mice in the presence of cancer. Cancers in WT mice were not only more advanced but demonstrated Paneth cell metaplasia, a distinguishing histologic finding in IBD in the distal colon [20, 21] that is also noteworthy because Paneth cells are a recognized source of *Ang4* [17].

A growing body of evidence supports the concept that angiogenin is dynamically regulated in colitis. While prior studies only evaluated colitis following a single DSS cycle [6, 7], we demonstrated findings that persisted through three cycles of DSS, which more closely mimics chronic inflammation. The upregulation of *Ang1* corresponded with a significant decrease in the severity of colitis, as assessed by DAI, the endoscopic appearance, histologic analysis, and by inflammatory cytokine expression. The differences seen during both acute colitis and recovery suggests that *Ang1* upregulation plays an important role in this response, particularly in the recovery phase of colitis. While this result appears to contradict previously published data [6] showing reduced angiogenin levels in the colon tissue of mice following 2.5% DSS, this may have been affected by the location of the tissue sample (proximal vs. distal). In human patients with IBD, the acuity and chronicity of colitis in the human patients was not well described [6]. This difference may also be secondary to epithelial cell loss leading to differences in endoscopic tissue sampling, and in any case highlights the need for further investigation [13].

Important questions remain regarding how angiogenin contributes to both the counter-regulatory response to colitis and the development of colitis-associated cancer. As antimicrobial peptides, we hypothesize that angiogenin-1 and angiogenin-4may regulate these processes through alterations in the gut microbiome. There has been considerable interest, and a growing body of evidence, supporting the link between the microbiome, IBD, and cancer. The gut microbial dysbiosis that characterizes IBD has been associated with altered function of antimicrobial peptides [13, 22, 23]. Particularly in the colon, where the mucosal surface and gut microbiota are in such close proximity, the regulation of that interface is critically important to GI function in both health and disease. In fact, 16S rRNA sequencing of stool comparing WT and *Ang1*-KO mice at baseline identified significant community level differences that support this hypothesis. Given the absence of *Ang1* and the 12-fold upregulation of *Ang4* at baseline in *Ang1*-KO mice, both *Ang1* and *Ang4* may be contributing to these microbial changes. Also notable is that despite repeated exposure to DSS, the distinct differences in community composition were sustained during recovery from colitis and in the presence of cancer. We hypothesize that these durable effects may be the mechanism by which angiogenin regulates chronic colitis as well as colitis-associated cancer. This is a focus of future investigations.

In conclusion, *Ang1*-KO mice develop more severe colitis but fewer tumors in the AOM-DSS model. This is supported by experiments utilizing *Ang1*-KO mice with an analysis of *Ang1* and *Ang4* expression, inflammatory cytokine mRNA expression and immunohistochemical analysis of colonic tissue. Taken together, these findings suggest that angiogenin subtypes may play an important role in the counter-regulatory response to colitis and the development of colitis-associated cancer. These findings may serve as a foundation for novel therapeutic approaches in the management of IBD that target angiogenin signaling.

## Supporting information

S1 Checklist The ARRIVE guidelines 2.0: Author checklist. (PDF)

S1 Data (TIF)
