# Supplementary figures and images for: Ang1 and Ang4 differentially affect colitis and carcinogenesis in an AOM-DSS mouse model
Source: PLoS One. 2023 Mar 7;18(3):e0281529. doi: 10.1371/journal.pone.0281529 (PMC9990929; doi:10.1371/journal.pone.0281529)

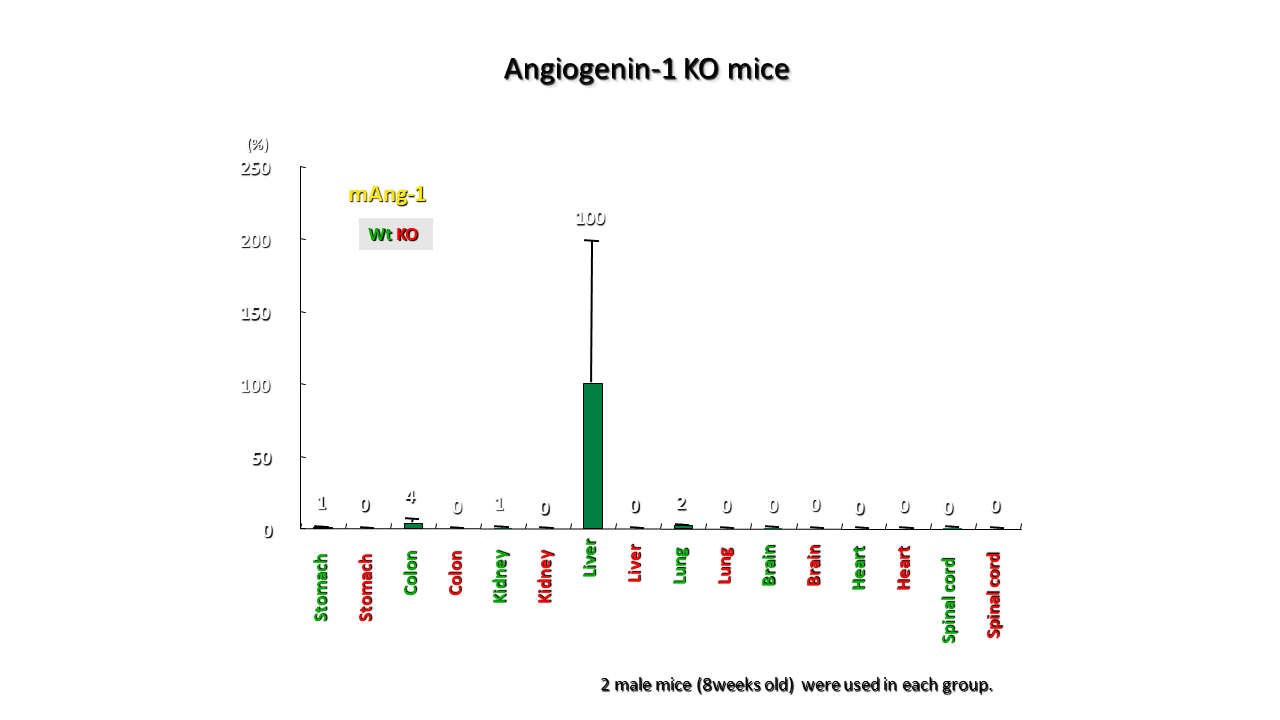

Supplement: S1 Data — (TIF) [file pone.0281529.s002.tif]
